# Synthesis and Electrochemical Properties of Two-Dimensional RGO/Ti_3_C_2_T*_x_* Nanocomposites

**DOI:** 10.3390/nano8020080

**Published:** 2018-01-31

**Authors:** Changjie Shen, Libo Wang, Aiguo Zhou, Bo Wang, Xiaolong Wang, Weiwei Lian, Qianku Hu, Gang Qin, Xuqing Liu

**Affiliations:** 1 School of Materials Science and Engineering, Henan Polytechnic University, Jiaozuo 454000, Henan, China; shenchangjie92@163.com (C.S.); zhouag@hpu.edu.cn (A.Z.); wangxiaolong@163.com (X.W.); WWL2116060@163.com (W.L.); hqk@hpu.edu.cn (Q.H.); clqingang@126.com (G.Q.); 2 State Key Laboratory of Solid Lubrication, Lanzhou Institute of Chemical Physics, Chinese Academy of Sciences, Lanzhou 730000, China; bowang@licp.cas.cn; 3 School of Materials, University of Manchester, Oxford Road, Manchester M13 9PL, UK; xuqing.liu@manchester.ac.uk

**Keywords:** MXene, graphene, exfoliation, nanocomposite, lithium-ion battery

## Abstract

MXene is a new type of two-dimensional layered material. Herein, a GO/Ti_3_C_2_T*_x_* nanocomposite was prepared by a simple liquid phase method, and the obtained GO/Ti_3_C_2_T*_x_* was transformed into RGO/Ti_3_C_2_T*_x_* under high temperature with Ar/H_2._ The prepared samples were characterized using X-ray diffraction (XRD), Raman measurement, scanning electron microscopy (SEM), energy disperse spectroscopy (EDS), and X-ray photoelectron spectroscopy (XPS). As an electrode material in lithium-ion batteries, the RGO/Ti_3_C_2_T*_x_* nanocomposite exhibited an excellent electrochemical performance and an excellent rate performance. Compared to pure Ti_3_C_2_T*_x_*, the nanocomposite had a better reversible capacity at different current densities and had no attenuation after 200 cycles, which is one time higher than pure Ti_3_C_2_T*_x_*. The improvement in the specific capacity was due to the excellent electrical conductivity and the unique structure of RGO, in which a charge transfer bridge was built among the Ti_3_C_2_T*_x_* flakes. Such a bridge shortened the transmission distance of the electrons and ions and effectively controlled the restacking of the laminated materials.

## 1. Introduction

In recent years, two-dimensional (2D) materials have become an important field of research because of their unique properties and have attracted an increasing amount of attention in materials science [1,2,3,4,5,6]. Graphene-based materials are those that include graphene [7] or graphene-like materials, which have a structure like graphene but contain other elements and compounds. For example, MXene contains Ti_3_C_2_T*_x_* and Ti_2_CT*_x_* [8,9]. MXene are a new kind of layered 2D transition metal carbides and nitrides and are prepared from a MAX phase by Naguib et al. [9] in 2011. The general formula of the MAX phase is M*_n_*_+1_AX*_n_* (*n* = 1, 2, 3,…), where M represents transition metal elements (M = Ti, Sr, V, Cr, Ta, Nb, Zr, Mo, and Hf), A represents Group IIIA or IVA elements (A = Al, Ga, In, Ti, Si, Ge, Sn, and Pb) and X represents C or/and N elements [10,11,12]. The A element is selectively etched from the MAX phase using hydrofluoric acid (HF) [9,13] or a mixture of fluoride salt and hydrochloric acid (HCl) [14] to produce MXene, which are known to possess graphene-like 2D structures. It is more precise to denote these compounds as M*_n_*_+1_X*_n_*. Thus far, more than 70 kinds of MAX phase compounds have been reported [15], but until now only the following families have been successfully prepared: Ti_3_C_2_, Ti_2_C, (Ti_0*.*5_Nb_0*.*5_)_2_C, (V_0*.*5_Cr_0*.*5_)_3_C_2_, Ti_3_CN, Ta_4_C_3_, Nb_2_C, V_2_C, and Nb_4_C_3_ [13,16]. Because of their unique structures, MXene has attracted considerable attention in many fields, such as adsorption materials [17,18,19], lithium-ion batteries [20,21,22,23,24,25,26], hydrogen storage materials [27], high capacitor electrode materials [28,29,30,31,32], and additives for polymer composites [33,34,35].

MXene nanoparticles have a 2D lamellar structure. As the most extensively studied MXene nanomaterial, Ti_3_C_2_T*_x_* is synthesized by etching Ti_3_AlC_2_ with hydrofluoric acid or a mixed solution of fluoride salts and hydrochloric acid. MXene nanosheets, owing to the strong van der Waals interaction between adjacent layers, will inevitably self-stack in the drying process. The restacking 2D sheets MXene have a limited electrolyte-accessible surface area, which leads to an insufficient use of the properties of MXene. This problem can be solved by creating an open structure that can provide more space for electrode materials to come into contact with the electrolyte by introducing spacers between the MXene layers, such as carbon nanotubes and graphene [36,37]; combined with other conductive materials, these material can alleviate the restacking and reduce the volume change during the charge/discharge process [38,39]. Liu et al. [40] reported that the Ti_3_C_2_/CNTs nanocomposite showed a capacity of 428.1 mAh/g at 0.5 C. Liang et al. [41] tested interwoven MXene nanosheets/carbon-nanotube Composites as Li-S cathode hosts, and a stable performance was obtained over long cycles with only 0.043% decay.

In this paper, we report a new method by which an individual Ti_3_C_2_T*_x_* nanolayer can be obtained. The obtained mixture of GO/Ti_3_C_2_T*_x_* can be further transformed into an RGO/Ti_3_C_2_T*_x_* nanocomposite by a simple reduction method under high temperature with Ar/H_2_, and using the RGO/Ti_3_C_2_T*_x_* nanocomposite as the anode in lithium-ion batteries has a much higher electrochemical performance than pure Ti_3_C_2_T*_x_*.

## 2. Experimental Sections

### 2.1. Preparation of Ti_3_C_2_T_x_

Ti_3_AlC_2_ is the precursor of Ti_3_C_2_T*_x_* and was synthesized in our previous report [34]. Ti_3_AlC_2_ powders were obtained from a mixture of Ti powders, Al powders, and graphite in a molar ratio of 3:1.1:2 (Beijing Xingrongyuan Technology Co. Ltd., Beijing, China), and the mixture of powders was then sintered at 1400 °C for 2 h in an argon atmosphere using a tube furnace (Luoyang shenjia kiln Co. Ltd., Luoyang, China). Then, 5 g of the Ti_3_AlC_2_ powders (400 mesh, ≤37 μm) were immersed in 100 mL of a mixed solution of HCl and LiF and stirred for 48 h at 60 °C. The mixed solution was then centrifuged and washed with deionized water until the supernatant liquid was neutral. Finally, the sample was dried in a vacuum oven at 70 °C for 12 h.

### 2.2. Preparation of GO/Ti_3_C_2_T_x_ and RGO/Ti_3_C_2_T_x_ Nanocomposites

GO was synthesized with expanded graphite by a modified Hummers method [42]. Firstly, 0.1 g of GO (a mass ratio of 20%) is dispersed in deionized water and using ultrasound separation for 30 min to spread them evenly. Then, 0.5 g of Ti_3_C_2_T*_x_* was added to the solution via ultrasonic shock for 30 min until a stable solution was formed, and was kept for 12 h. The obtained sample was frozen with liquid nitrogen and dried using a freeze dryer (BioSafer Technology Co. Ltd., Guangzhou, China). The as-prepared powder was the GO/Ti_3_C_2_T*_x_* nanocomposite. In order to obtain the RGO/Ti_3_C_2_T*_x_* nanocomposite, GO/Ti_3_C_2_T*_x_* was sintered using a tube furnace at 450 °C for 4 h with a mixture of argon (98%) and hydrogen (2%).

### 2.3. Material Characterization

The crystal structures and morphologies of the samples were measured using X-ray powder diffraction (XRD, Rigaku SmartLab X-ray diffractometer with Cu Kα radiation, accelerating voltage = 40 kV, Tokyo, Japan), scanning electron microscopy (SEM, Merlin Compact, Carl Zeiss NTS GmnH accelerating voltage = 15 kV, Jena, Germany), and energy dispersive spectroscopy (EDS, Oxford instruments Co. Ltd., Oxford, UK). Raman spectra of the powder samples were measured on a LabRAM HR800 Raman microscope (Edison, NJ, USA) with a laser excitation wavelength of 532 nm. The chemical states of the elements in the samples were characterized using X-ray photoelectron spectroscopy (XPS) on a PHI-5702 multifunctional X-ray photoelectron spectrometer (Perkin–Elmer, Waltham, MA, USA). Mg Kα radiation was used as the excitation source. The binding energies of the target elements were determined with a pass energy of 29.35 eV and a resolution of about 0.3 eV.

### 2.4. Electrochemical Measurement

To investigate the electrochemical properties of the RGO/Ti_3_C_2_T*_x_* nanocomposite, experiments were carried out in a standard LIR2016-type coin cell. The working electrodes were made by grind the mixture of 80 wt % sample materials, 10 wt % polyvinylidenefluoride (PVDF), and 10 wt % acetylene black. Then a solution of *N*-methyl-2-pyrrolidone (NMP) was added and stirred for several minutes. Finally, the mixture slurry was coated on Cu foil and dried in a vacuum oven at 110 °C for 12 h. The dried electrode sheets were punched into discs (14 mm) and then assembled into a coin cell in an argon-filled glove box using lithium metal as the cathode electrode, and 1 M LiPF_6_ solution in a mixture of ethylene carbonate (EC)/dimethyl carbonate(DMC)/ethylmethyl carbonate (EMC) in a 1:1:1 volume ratio was used as the electrolyte. A microporous membrane was used as the separator. The coin cell was tested on a Xinwei battery tester; the electrochemical window was 0.01–3.0 V, and the current densities were 50, 100, 400, 800, and 1000 mA/g. The electrochemical impedance spectroscopy (EIS) was characterized using an electrochemical workstation (Parstat 2273, Princeton, NJ, USA) with a range from 100 kHz to 50 mHz.

## 3. Results and Discussion

**Figure 1:**
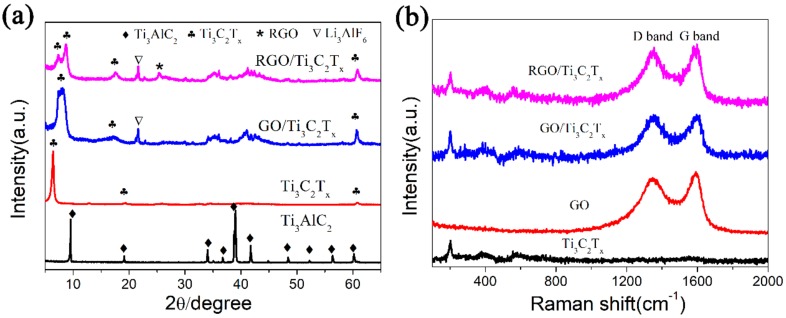
(**a**) X-ray diffraction (XRD) patterns and (**b**) Raman spectrum of Ti_3_AlC_2_, Ti_3_C_2_T*_x_*, and GO/Ti_3_C_2_T*_x_*, and RGO/Ti_3_C_2_T*_x_* nanocomposites.

The X-ray diffraction (XRD) patterns of Ti_3_AlC_2_, Ti_3_C_2_T*_x_*, and GO/Ti_3_C_2_T*_x_* and RGO/Ti_3_C_2_T*_x_* nanocomposites are shown in Figure 1a. As seen in the XRD patterns, the characteristic peaks of Ti_3_AlC_2_ disappeared, while a relatively strong characteristic peak of Ti_3_C_2_T*_x_* can be found at about 6.5°. After the combination of Ti_3_C_2_T*_x_* with GO, the characteristic peaks of Ti_3_C_2_T*_x_* were detected in the GO/Ti_3_C_2_T*_x_* nanocomposite; the (002) diffraction peak of Ti_3_C_2_T*_x_* was broader, and the full width at half maximum (FWHM) increased, which means that there was minimal stacking and a random arrangement of Ti_3_C_2_T*_x_* nanolayers. After heat treatment at high temperature in Ar/H_2_, a double peak appeared at small angles in the XRD pattern of RGO/Ti_3_C_2_T*_x_*, and this was mainly attributed to the different inter-layer spacing; the two peaks correspond to Ti_3_C_2_T*_x_*. Meanwhile, a weak peak around 2θ = 25° was observed, indicating that GO nanosheets were reduced to form RGO. Besides the diffraction peaks of Ti_3_C_2_T*_x_* and RGO, Li_3_AlF_6_ was also found. In the XRD pattern of pure Ti_3_C_2_T*_x_*, there were no peaks corresponding to Li_3_AlF_6_. This was because Li_3_AlF_6_ was hidden between the Ti_3_C_2_T*_x_* layers and exposed only after the exfoliation of Ti_3_C_2_T*_x_* into individual nanolayers. The Raman spectra were measured to further confirm the structure and composition of the as-prepared samples. As shown in Figure 1b, compared to pure Ti_3_C_2_T*_x_*, the samples of both GO/Ti_3_C_2_T*_x_* and RGO/Ti_3_C_2_T*_x_* had two distinct peaks at 1350 cm^−1^ (D band) and 1590 cm^−1^ (G band), and these findings are consistent with the literature [43]. The results indicate that RGO and Ti_3_C_2_T*_x_* were successfully combined.

**Figure 2:**
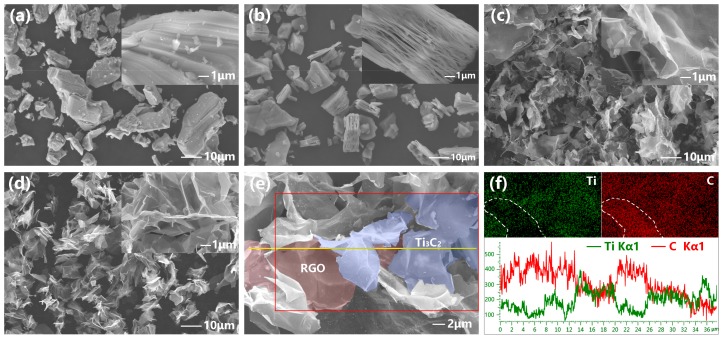
Scanning electron microscopy (SEM) image of Ti_3_AlC_2_ (**a**), Ti_3_C_2_T*_x_* (**b**), GO/Ti_3_C_2_T*_x_* (**c**), and RGO/Ti_3_C_2_T*_x_* (**d**). Element area profile and linear scanning of RGO/Ti_3_C_2_T*_x_* (**e**,**f**).

Figure 2a,b shows the scanning electron microscopy (SEM) images of Ti_3_AlC_2_ and Ti_3_C_2_T*_x_*. It can be clearly seen that Ti_3_C_2_T*_x_* had a typical layer structure with a broad particle size distribution. The size of the Ti_3_C_2_T*_x_* particles was on the micrometer scale. The gap between Ti_3_C_2_T*_x_* nanolayers is about several hundred nanometers. Figure 2c,d are SEM images of the GO/Ti_3_C_2_T*_x_* and RGO/Ti_3_C_2_T*_x_* nanocomposites. There were no lamellar-structure microparticles of Ti_3_C_2_T*_x_*, and there was clearly a typical individual flake shape. Additionally, small amounts of nanoparticles were found, and there were Li_3_AlF_6_ impurities, according to the XRD analysis. The red rectangle and yellow line in Figure 2e represent the surface and linear scanning ranges, respectively, and the results are shown in Figure 2f. The light and dark regions indicate the elemental contents. The region in the lower left corner of the Ti mapping is darker, while in the C mapping it is light, which means that graphene was located at this position. In combination with the linear scanning of Ti and C, the distributions of RGO and Ti_3_C_2_T*_x_* are shown in Figure 2e with different colors. From this, it was determined that the multilayers of Ti_3_C_2_T*_x_* were successfully exfoliated, and the sheets were separated into a single-layer open structure.

**Figure 3:**
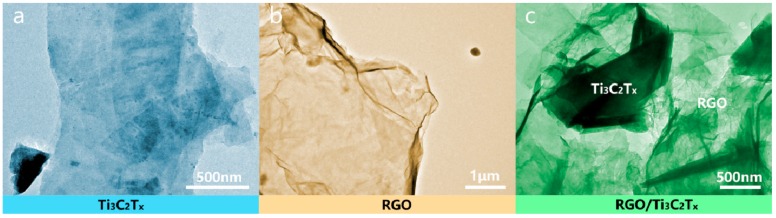
TEM image of Ti_3_C_2_T*_x_* (**a**), RGO (**b**), and RGO/Ti_3_C_2_T*_x_* (**c**).

The transmission electron microscopy (TEM) image was used to further characterize the microstructure of the nanomaterials, and the TEM result is shown in Figure 3. As shown in Figure 3a, the layer structure of Ti_3_C_2_T*_x_* nanosheets has stacked together; in Figure 3b, there is a typical graphene sheet structure. In the TEM image of RGO/Ti_3_C_2_T*_x_* (Figure 3c), the nanocomposite has been stripped into a more fragile sheet; which is consistent with the SEM results.

**Figure 4:**
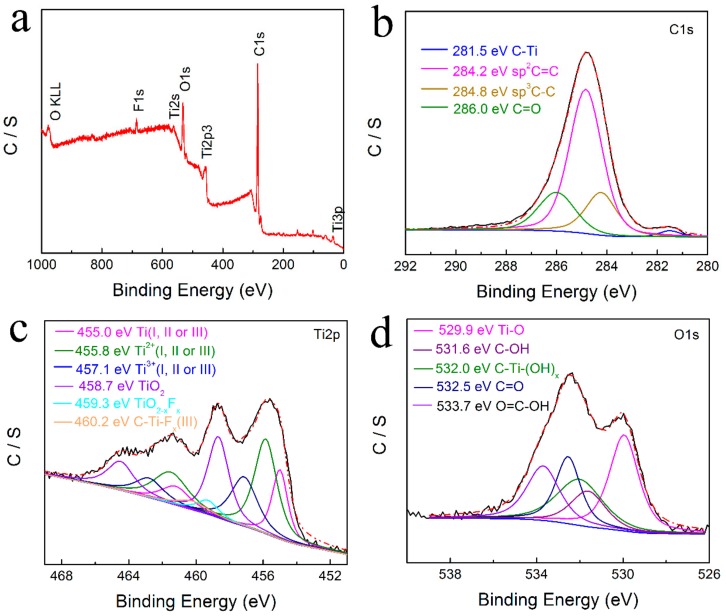
The survey spectrum (**a**) and high-resolution XPS spectra of the C1s (**b**), Ti2p (**c**), and O1s (**d**) peaks of the RGO/Ti_3_C_2_ nanocomposite.

The surface of the RGO/Ti_3_C_2_T*_x_* nanocomposite was analyzed using X-ray photoelectron spectroscopy (XPS). The survey spectra (Figure 4a) and the high-resolution XPS spectra of Ti2p, C1s, and O1s of the RGO/Ti_3_C_2_T*_x_* sample are shown in Figure 4b–d. The Ti2p spectrum can be deconvoluted into six peaks corresponding to Ti atoms (455.0, 455.8, and 457.1 eV), Ti–O (458.7 eV), TiO_2-*x*_F*_x_* (459.3 eV), and C–Ti–F*_x_* (460.2 eV), and this is consistent with the literature [44]. The C1s XPS spectrum of the RGO/Ti_3_C_2_ nanocomposite (Figure 4b) was fitted using four peaks. The dominant peak corresponds to the C–C bond, accompanied by three minor peaks at 286.0, 284.2, and 281.5 eV which are assigned to the C=O, C=C, and C–Ti bonds, respectively [45,46,47]. The O1s peak can be deconvoluted into five symmetrical peaks. The fitting peaks around 529.9, 531.6, 532.0, 532.5, and 533.7 eV are attributed to Ti–O, C–OH, C–Ti–(OH)*_x_*, C=O, and O=C–OH [44,48]. The results show that Ti_3_C_2_T*_x_* and RGO formed a hybrid structure composite, and this is in good agreement with the SEM images and the XRD pattern.

**Figure 5:**
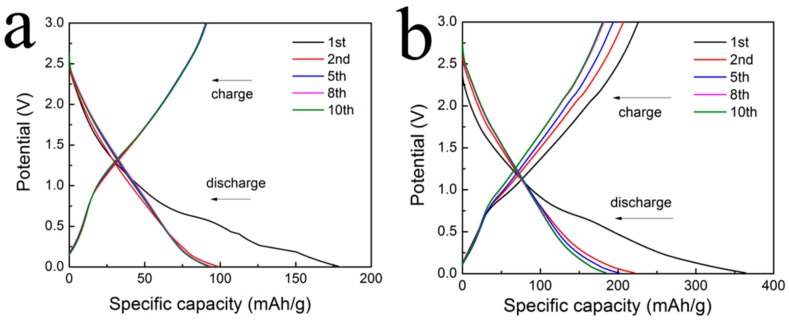
Charge–discharge profiles of (**a**) Ti_3_C_2_T*_x_* and (**b**) the RGO/Ti_3_C_2_T*_x_* nanocomposite electrode at different cycles with a current density of 50 mA/g.

**Figure 6:**
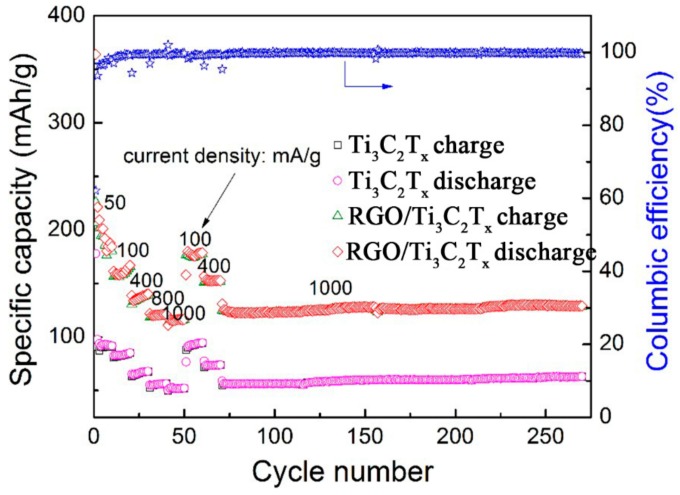
Rate performance of Ti_3_C_2_T*_x_* and the RGO/Ti_3_C_2_T*_x_* nanocomposite at different current densities.

The electrochemical properties of the RGO/Ti_3_C_2_T*_x_* nanocomposite were tested using a LIR2016 coin-type cell. Galvanostatic charge-discharge (GCD) cycling and electrochemical impedance spectroscopy (EIS) were also carried out. Specific capacity is usually one of the most important parameters for evaluating the performance of electrode materials. The charge-discharge profiles of Ti_3_C_2_T*_x_* and the RGO/Ti_3_C_2_T*_x_* nanocomposite as the activities for lithium-ion battery (LIB) anode at different cycles with a current density of 50 mA/g in the voltage range from 0.01 to 3.0 V (vs. Li^+^/Li) are shown in Figure 5, respectively. The first discharge capacities of Ti_3_C_2_T*_x_* and the RGO/Ti_3_C_2_T*_x_* nanocomposite are 178 and 364 mAh/g, respectively. The first discharge capacity of the RGO/Ti_3_C_2_T*_x_* nanocomposite was much higher than the theoretical capacity of Ti_3_C_2_T*_x_* (~260 mAh/g) [49]. The stabilities and rate performances of Ti_3_C_2_T*_x_* and the RGO/Ti_3_C_2_T*_x_* nanocomposites at various current densities are shown in Figure 6. The first charge-discharge coulombic efficiencies for Ti_3_C_2_T*_x_* and the RGO/Ti_3_C_2_T*_x_* composite electrodes are 53% and 62%. The irreversible capacity and the low efficiency of the first cycle of the charge-discharge behavior are mainly ascribed to the irreversible electrolyte reduction on the layers of the nanocomposite and to the formation of a solid electrolyte interface (SEI) [14,50]. In subsequent cycles, the discharge capacities of Ti_3_C_2_T*_x_* were 98, 82, 65, 55, and 50 mAh/g at current densities of 50, 100, 400, 800, and 1000 mA/g, respectively; for the RGO/Ti_3_C_2_T*_x_*nanocomposite, at the same current densities, the discharge capacities in subsequent cycles were 221, 161, 138, 122, and 111 mAh/g, respectively. These results show that the RGO/Ti_3_C_2_T*_x_* nanocomposite had better electrochemical performance than Ti_3_C_2_T*_x_* and benefited from the improved conductivity and the open structure. The open structure increases Li ion access to active sites and connected the layered Ti_3_C_2_T*_x_* [38,39,51].

**Figure 7:**
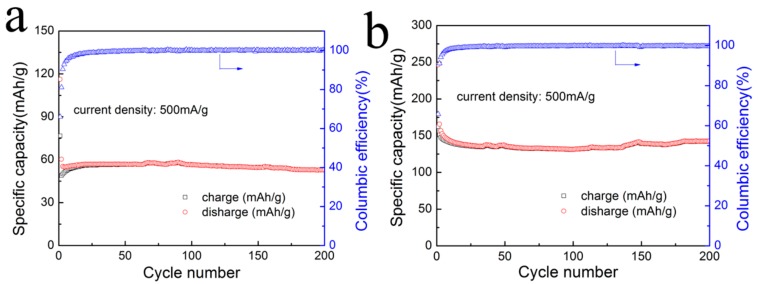
Cycling performance of (**a**) Ti_3_C_2_T*_x_* and (**b**) the RGO/Ti_3_C_2_T*_x_* nanocomposite at a current density of 500 mA/g.

In addition, in terms of cycle stability, the results of Ti_3_C_2_T*_x_* and the RGO/Ti_3_C_2_T*_x_* nanocomposite at a current density of 500 mA/g for 200 cycles are shown in Figure 7. At the same current density, the RGO/Ti_3_C_2_T*_x_* nanocomposite had a higher capacity than Ti_3_C_2_T*_x_*; in particular, a specific capacity of 140 mAh/g was obtained, which is much higher than that of pure Ti_3_C_2_T*_x_* (53 mAh/g). Compared with a previous report on SnO_2_-GNS materials [52], the capacity of the RGO/Ti_3_C_2_T*_x_* material shows almost no attenuation. The columbic efficiencies of Ti_3_C_2_T*_x_* and the RGO/Ti_3_C_2_T*_x_* nanocomposite are both close to 100%. In contrast, the RGO/Ti_3_C_2_T*_x_* nanocomposite had a higher capacity than that of Ti_3_C_2_T*_x_* at the same current densities. Furthermore, it was also found that, after a number of cycles, the capacity of the RGO/Ti_3_C_2_T*_x_* nanocomposite increased slowly with cycling. This result was possibly because of the specific structures that had loose morphologies with minimal restacking of Ti_3_C_2_T*_x_* nanolayers, and this facilitated the electrochemical activation process and improved the electrochemical performance [53].

**Figure 8:**
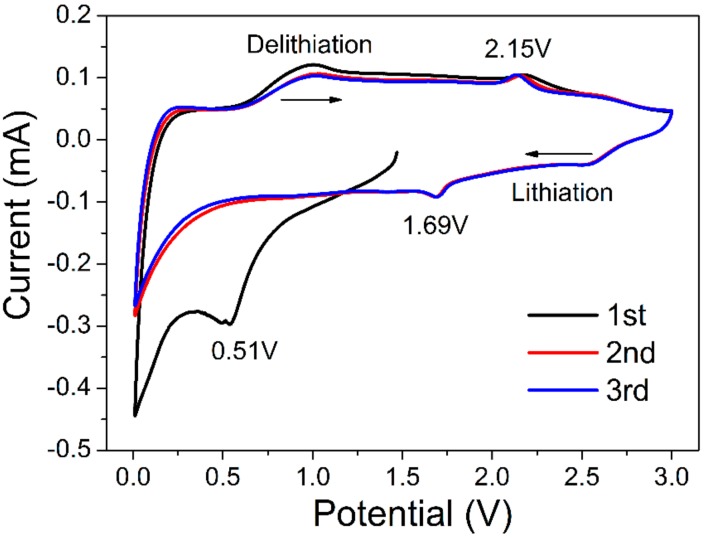
Cyclic voltammetry (CV) curves of the RGO/Ti_3_C_2_T*_x_* nanocomposite from 3.0 to 0.01 V at a scan rate of 0.2 mV/s.

Figure 8 shows the cyclic voltammetry (CV) test results of the RGO/Ti_3_C_2_T*_x_* nanocomposite. In the process of first lithiation, a peak around 0.51 V was observed and was attributed to the formation of a solid electrolyte interphase (SEI) [48]; the peak at 1.69 V was caused by the trapping of Li^+^ between the sheets in the electrode materials [23,54]. In the process of delithiation, a peak around 2.15 V was observed and corresponded to the extraction of Li^+^ from the layer electrode. From the reported literature [43], in the pure graphene, there was no obvious redox peaks, excluding the reduced peaks at low voltage.

**Figure 9:**
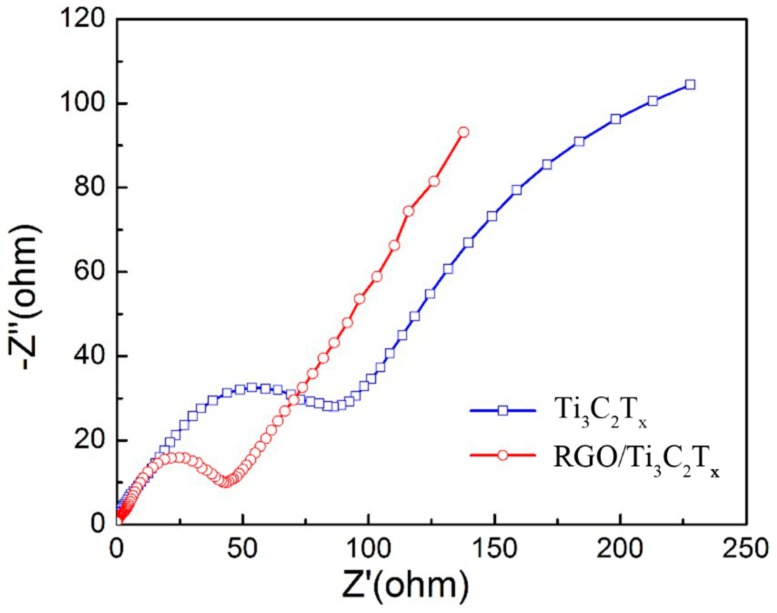
Electrochemical impedance spectroscopy (EIS) plots of the Ti_3_C_2_T*_x_* and RGO/Ti_3_C_2_T*_x_*.

The EIS of Ti_3_C_2_T*_x_* and RGO/Ti_3_C_2_T*_x_* are shown in Figure 9. The EIS spectrum consists of a semi-circle and a sloped line. The formation of the semi-circle was attributed to the movement of the lithium ions and to the charge transfer through the SEI films [12,40]. The diameter of the semi-circle was related to the charge transfer resistance at the electrode. Comparing the Nyquist plots of the two different samples, the RGO/Ti_3_C_2_T*_x_* nanocomposite had a relatively small semicircular diameter, which indicates that the RGO/Ti_3_C_2_T*_x_* nanocomposite had a much lower charge transfer resistance than pure Ti_3_C_2_T*_x_*. This was mainly attributed to the addition of RGO, which provided many effective conductive pathways and improved the electron transfer between the Ti_3_C_2_T*_x_* flakes. Additionally, the exfoliation of 2D Ti_3_C_2_T*_x_* materials effectively increased the contact area between Ti_3_C_2_T*_x_* and the electrolyte, which may decrease resistance and promote electron transfer. Finally, the hybrid structure provided more active fresh sites or Li ion diffusion channels. The above analyses from the electrochemical tests confirmed that the RGO/Ti_3_C_2_T*_x_* nanocomposite has excellent reversibility and rate stability.

## 4. Conclusions

In this study, 2D/2D RGO/Ti_3_C_2_T*_x_* nanocomposites were successfully prepared using a simple method and was used as an electrode in lithium-ion batteries. The RGO/Ti_3_C_2_T*_x_* nanocomposite had a hybrid structure and exhibited better performance than pure Ti_3_C_2_T*_x_*. In the nanocomposite, RGO helped develop paths for fast electron transport, in which a charge transfer bridge was built among the Ti_3_C_2_T*_x_* flakes. Such a bridge shortened the transmission distance of the electrons and ions and effectively controlled the restacking of the laminated materials. Furthermore, the RGO/Ti_3_C_2_T*_x_* nanocomposite exhibited a stable capacity of 125 mAh/g and maintained a relatively high coulomb efficiency at a current density of 1000 mA/g for 200 cycles.
